# Antitumoral immunity induced by gel ethanol ablation to treat unresectable colorectal cancer metastases in the liver

**DOI:** 10.1371/journal.pone.0347625

**Published:** 2026-04-22

**Authors:** Jeffrey Yang, Robert Morhard, Hannah Huth, Baktiar Karim, John W. Karanian, Bradford J. Wood, Andrew S. Mikhail, Jenna L. Mueller

**Affiliations:** 1 Fischell Department of Bioengineering, University of Maryland, College Park, Maryland, United States of America; 2 Center for Interventional Oncology, Radiology and Imaging Sciences, Clinical Center, National Institutes of Health, Bethesda, Maryland, United States of America; 3 Molecular Histopathology Laboratory, Laboratory Animal Science Program, Frederick National Laboratory for Cancer Research, National Institutes of Health, Frederick, Maryland, United States of America; 4 Marlene and Stewart Greenebaum Cancer Center, University of Maryland School of Medicine, Baltimore, Maryland, United States of America; Al-Azhar University, EGYPT

## Abstract

Colorectal cancer (CRC) is the second leading cause of cancer-related deaths worldwide. A quarter of CRC patients develop liver metastases. Treatment options for liver metastases include surgically removing the tumors or undergoing liver transplantation; however, many patients are ineligible for these treatments due to severe extrahepatic disease or lack of suitable donors. Radiofrequency ablation offers an alternative local treatment modality for resolving CRC liver metastases and is known to generate antitumoral effects to stunt contralateral tumor growth. However, radiofrequency ablation is not suitable for tumors situated near critical structures or large blood vessels. Ethanol ablation is an alternative treatment option where pure ethanol is directly injected into tumors to induce necrosis and is unhindered by the drawbacks from radiofrequency ablation. The addition of ethyl cellulose with ethanol (EC-ethanol) enhances its retention within tissue and subsequently improves tumor ablative efficacy. However, the antitumoral response following EC-ethanol ablation in CRC tumors is poorly understood. Thus, we utilized a CRC murine model to investigate the immune effects following EC-ethanol treatment. Studies in the single flank model demonstrated up to a 27-fold increase in IL-6 and KC/GRO pro-inflammatory cytokines within 6 hours post-treatment compared to sham treatments, along with a 4-fold increase in target-tissue necrosis and increased cytotoxic T-cells within the vicinity of the ablation zone over 7 days. Studies in the bilateral flank tumor model demonstrated that EC-ethanol ablation on the primary tumor resulted in a 1.6-fold increase in cytotoxic T-cells within the contralateral tumor after 7 days compared to the sham control group. Combining EC-ethanol treatment with radiofrequency ablation resulted in a more pronounced, 2-fold increase in cytotoxic T-cells within the contralateral tumor. Altogether, these results suggest that EC-ethanol potentiates antitumoral effects in CRC tumors and is a strong therapeutic candidate for treating CRC patients worldwide.

## Introduction

Colorectal cancer (CRC) is the second leading cause of cancer-related mortality worldwide, and this burden is expected to double by 2040 [1]. Nearly 1 in 4 patients with CRC are diagnosed with metastatic disease in the liver [2–5] and consequently have a 5-year overall survival rate below 20% [6,7]. Treatments of CRC liver metastases include surgical resection where tumors are physically excised from the affected liver tissue [8] and, in highly selective cases, liver transplantation, which involves replacing the diseased liver with a healthy liver from a donor. These interventions improve 5-year overall survival rates to as high as 80% [9,10]. However, 15–30% of patients are ineligible for these treatments due to extensive extrahepatic disease, a paucity of suitable organ donors, or insufficient postoperative liver volume and subsequent hepatic functionality [11–13].

Local ablative therapies are a well-established alternative treatment option for patients with unresectable CRC liver metastases. Radiofrequency ablation (RFA) is one of the most commonly used liver ablative therapies; it generates heat via a high frequency alternating current delivered through needle electrodes inserted into tumors, resulting in thermal damage. A randomized, controlled, phase III non-inferiority clinical trial found no difference in overall survival and local tumor control, as well as fewer adverse events for RFA compared to surgical resection for CRC metastases up to 3 cm [14]. RFA was also shown to induce the creation of a highly immunogenic microenvironment [15,16] further enhancing its ablative efficacy on CRC metastases. However, RFA may not be suitable when there is a risk of thermal injury to nearby critical tissues or a substantial heat-sink effect that diminishes ablative efficacy near large blood vessels [17]. An alternative ablative therapy not hindered by these drawbacks is ethanol ablation, a chemical ablation method in which pure ethanol is directly administered to tumors to induce necrosis [18]. The combination of ethanol ablation and RFA has been shown to enhance ablative effectiveness in liver tumors, as performing ethanol ablation before RFA occludes the vasculature, thereby diminishing the heat-sink effect and enhancing RFA’s ablative efficacy [19,20].

Ethanol ablation does, however, suffer from leakage into surrounding tissues, which can reduce efficacy or cause adverse off-target tissue damage [21]. To resolve this issue, we added the polymer ethyl cellulose (EC) to ethanol, which upon injection into tissue forms a gel depot that improves retention of ethanol at the injection site [22]. An initial study in which 3% EC-ethanol (EC:ethanol, w:w) was injected into a preclinical model of oral carcinoma demonstrated complete tumor regression after 7 days, compared with pure ethanol injections, which were ineffective in eradicating the tumors [22]. The EC-ethanol formulation was further optimized in swine liver, where 6% EC-ethanol demonstrated improved injectate delivery and depot formation compared to 3% EC-ethanol and pure ethanol [23].

Recent evidence suggests that EC-ethanol, similar to other ablative therapies like RFA, may stimulate antitumor immune responses and fortify the effects of immunotherapy [15,16]. The treatment of a triple-negative metastatic breast cancer mouse model with EC-ethanol resulted in a significant 3-fold increase in recruitment of CD3 + T cells into the tumors after 30 days post-ablation [24]. In addition, EC-ethanol increased the responsiveness to anti-PD1 and anti-CTLA4 checkpoint inhibitor immunotherapies and improved overall survival of mice bearing 4T1 metastatic tumors after 60 days compared to EC-ethanol or checkpoint inhibition alone [24],showing the potential to use EC-ethanol as an adjuvant immunomodulatory therapy.

The objective of this study was to establish the immune effects following EC-ethanol ablation in CRC tumors. A highly immunogenic CRC murine model was selected as it had previously been used by our group to characterize the immune response generated by various ablation technologies [25]. Both single and bilateral flank tumor models were used to examine the local and systemic effects induced by EC-ethanol ablation. Tumor ablations was performed, and an immune investigation was conducted over 7 days to evaluate local and systemic immune effects. An RFA cohort was included as a positive comparator for the tumor ablation experiments, given its known ability to induce systemic antitumor effects [26]. Additionally, a combination cohort was included to establish the synergistic potential of EC-ethanol when used in combination with other established ablative therapies.

## Materials and methods

### Preparation of EC-ethanol solution

EC (Sigma Aldrich, St. Louis, MO, USA) was dissolved in 200 proof ethanol (The Warner Graham Company, Cockeysville, MD, USA) with a stir plate at room temperature, as previously described [27]. The ratio of EC to ethanol was 6% (EC:ethanol, w:w) for all experiments.

### Induction of CT26 tumors in mice for ablative treatment

**Fig 1:**
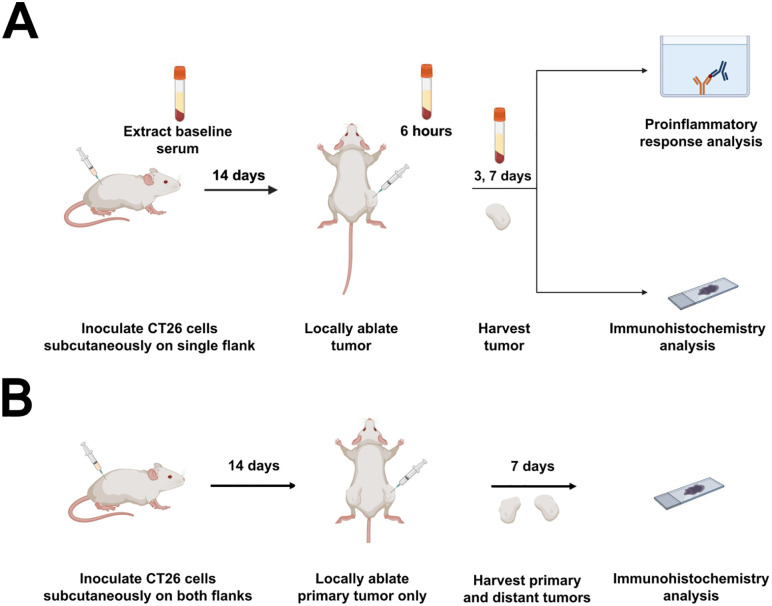
Study workflow and associated analyses. (A) Mice were inoculated on their right flank with CT26 cells and allowed to grow for 14 days. Tumors were then locally ablated. After 3 and 7 days post-treatment, the mice were sacrificed and tumors harvested for immunohistochemistry analysis. Serum was also collected at each time point for conducting cytokine analyses. (B) Mice were inoculated on both flanks with CT26 cells. After 14 days, the primary tumor was ablated. Primary and contralateral tumors harvested after 7 days post-treatment for immunohistochemical analysis. Created with Biorender.com.


This study was conducted under an animal use protocol approved by the Animal Care and Use Committee of the NIH Clinical Center (DRD 23–04). All procedures were performed in accordance with relevant animal welfare guidelines and regulations. **Fig 1** describes the study workflow and methods. CT26 murine CRC cells (American Type Culture Collection, Manassas, VA, USA) were maintained by culturing in phenol red-free RPMI-1640 medium (Gibco, Thermo Fisher Scientific, Waltham, MA, USA) containing 10% heat-inactivated fetal bovine serum (Gibco), 100 U/mL penicillin, 100 µg/mL streptomycin, and HEPES buffer (Quality Biological, Gaithersburg, MD, USA). For the single flank model (Fig 1A), female wild type BALB/c mice (Charles River Laboratories, Inc., Wilmington, MA, USA) between 6–8 weeks old were inoculated on their flank subcutaneously with 1.0 x 10^6^ cells suspended in 100 µL Hank’s Buffered Saline Solution (Gibco), as previously described [28]. The tumor volume (TV) and mouse body weight were measured and monitored every 2–3 days. The following formula was used to calculate TV:

$$TV=\frac{(major\;axis)\;x\;{\left(minor\;axis\right)}^{\text{2}}}{\text{2}}$$ (1)

For the bilateral tumor model (Fig 1B), mice were simultaneously inoculated with 1.0 x 10^6^ and 2.0 x 10^5^ CT26 cells on opposite flanks in order to generate primary tumors for subsequent treatment or sham procedures and contralateral untreated tumors, respectively.

### Ablation procedures

All procedures were performed under general isoflurane (1–3% v/v) anesthesia. Table 1 provides an overview of the treatment groups associated with each experiment. For the single flank study arm, when TV reached approximately 250 mm^3^, mice were divided into: 1) EC-ethanol ablation and 2) sham control cohorts (20 total mice, n = 10 for each cohort) to establish local antitumor effects induced by EC-ethanol. For the EC-ethanol cohorts, 60 µL of 6% EC-ethanol was intratumorally infused at the center of the tumor with a 27-gauge hypodermic needle at 30 mL/hr infusion rate using a programmable syringe pump, as previously described [29,30]. This delivery setup generated a subtherapeutic dosage on tumors to evaluate any induced immune effects within both treated and untreated tumor regions [31–33]. Sham procedures were performed by insertion of the needle at the center of the tumor without ethanol infusion, for the same duration as the treatment cohorts.

**Table 1:** Overview of ablation treatments used in each experiment and relevant endpoints.

Experiment	Animal model	Treatment (n = 10)	Termination	Relevant Endpoints
***1***	CT26 murine single flank	Sham	3 days post-treatment 7 days post-treatment	Pro-inflammatory cytokines in serum, immune cell profiling via immunohistochemistry analysis of tumors
		EC-ethanol		
***2***	CT26 murine bilateral flank	Sham	7 days post-treatment	Immune cell profiling via immunohistochemistry analysis of tumors
		EC-ethanol		
		RFA		
		Combination (EC-ethanol + RFA)		

For the bilateral tumor model, when the primary tumor reached approximately 250 mm^3^, mice were divided randomly into: 1) EC-ethanol ablation, 2) RFA, 3) EC-ethanol + RFA combination, and 4) sham control cohorts (40 total mice, n = 10 for each cohort) to evaluate antitumor effects induced by EC-ethanol in the contralateral tumor. Delivery of EC-ethanol was performed using the the same procedure described for the single flank study arm. For the RFA cohort, a 22-gauge cannula with a 2 mm uninsulated tip (Boston Scientific, Marlborough, MA, USA) connected to a radiofrequency lesion generator (Baylis Medical Company, Inc., Mississauga, ON, Canada) was inserted into the center of the primary tumor under ultrasound guidance, and heat at 90°C was applied for 90 seconds, as previously described [25]. For the combination cohort, the EC-ethanol treatment protocol proceeded first, followed immediately by RFA treatment. Performing the concomitant treatments in this order has been previously shown to produce larger areas of necrosis and improved stunting of tumor growth [34–36]. Buprenorphine at 0.05 mg/kg dosage was administered subcutaneously to all mice of both study arms immediately after treatment. All mice were monitored daily after treatments and were humanely euthanized if tumors reached a cumulative TV of 2000 mm^3^ before reaching the target time points of 3 days or 7 days for euthanasia. None of the tumors surpassed 2000 mm^3^, and all mice were euthanized by CO2 asphyxiation followed by cervical dislocation once 3 or 7 days post-treatment were reached.

### Serum cytokine analysis

Whole blood was collected from each mouse in the single flank model via submandibular bleeding at the following time points, as performed previously [25]: 1) the day of tumor inoculation to establish baseline readings [25], 2) six hours post-ablation [25], 3) 3 days post-ablation [25], and 4) 7 days post-ablation to explore the pro-inflammatory immune response. Serum was then promptly extracted and cryopreserved. All serum specimens were analyzed for pro-inflammatory cytokines via an electrochemiluminescence assay (V-PLEX Proinflammatory Panel 1 Mouse Kit, Meso Scale Diagnostics, LLC, Gaithersburg, MD, USA). Concentration values of each cytokine were normalized to values obtained from sera collected the day of tumor inoculation.

### Tumor histopathology analysis

Immediately after euthanasia, the tumors were excised and fixed in 10% neutral-buffered formalin (Azer Scientific, Morgantown, PA, USA), embedded in paraffin, and sectioned. 5 µm thick sequential sections were obtained from the center of each tumor and mounted onto glass slides. Then, sequential slides were stained with hematoxylin & eosin (H&E) and immunohistochemistry (IHC) staining for immune markers. Specifically, IHC staining included heat-induced epitope retrieval (Citrate) 20’ for CD8a (eBioscience Catalog #14-0195-82 rat IgG2a, 1:50). The Bond Polymer Refine Kit (minus PostPrimary Reagent) was used for both markers, along with a rabbit anti-rat secondary antibody for CD8a. Normal mouse spleen was used as positive control tissue for CD8a. For negative controls, isotype control antibodies replaced the primary antibodies.

All image analyses were performed in HALO software (Indica Labs, Alberquerque, NM, USA) in a blinded fashion by a veterinary pathologist (BK). The ablation zones were defined by the necrotic tissue resulting from the treatments. The margins of the ablation zone were defined as 400 µm concentric regions, which contained the areas containing positive stainings surrounding the ablation zone perimeter. Tumor margins were analyzed via concentric partitioning analysis using the Cytonuclear HALO image analysis platform. For sham treatments, margins surrounding necrotic pockets were delineated. For necrosis quantification, the percent tissue necrosis induced by the treatments was calculated by dividing the area of necrosis by the total tumor area in the H&E-stained slides and then multiplying by 100. For CD8a+ cytotoxic T-lymphocyte (CTL) quantification, the population density (i.e., positive cells per mm^2^) for a specific region (i.e., whole tumor, ablation zone, margins of the ablation zone) was calculated by dividing the total number of positively-stained cells within the specific region by the area of the specific region in the IHC-stained slides.

### Statistical analysis

Statistical analyses were performed using Graphpad Prism (GraphPad Software, San Diego, CA, USA). For the serum cytokine analysis and H&E analysis of necrosis for the single flank tumor study, an unpaired t-test was used to compare differences between cytokine levels induced by EC-ethanol and sham treatments at the same time point. Additionally, a repeated measures ANOVA was performed on the cytokine normalized concentrations throughout the treatment timeline to compare differences in cytokine levels from the initial time point within each treatment group. For all IHC plots, the Mann-Whitney U test was used to evaluate any significant differences in positive cells per mm^2^ between day 3 and day 7 time points for each treatment group. Additionally, for the bilateral flank tumor study, the Kruskal-Wallis non-parametric analysis of variance was used to compare significant differences in positive cells per mm^2^ between all treatment groups within the day 7 time point. A significance level of *p* = 0.10 was a*p*plied to reject the null hypothesis in all analyses.

## Results

### EC-ethanol treatment increased expression of pro-inflammatory cytokines

**Fig 2:**
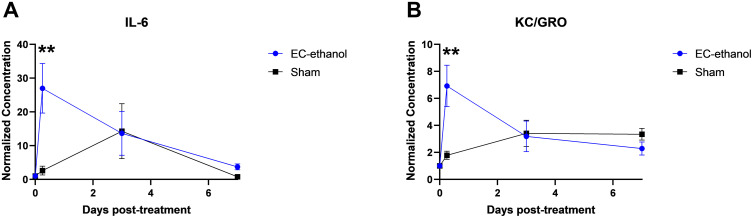
Pro-inflammatory cytokine expression induced by EC-ethanol ablation. The fold-change of (A) IL-6 and (B) KC/GRO over a period of 7 days (n = 8-10). Error bars are standard error of the mean (SEM). *P < 0.10, **P < 0.05, ***P < 0.01, ****P < 0.001.

Fig 2 shows the fluctuations in concentration for two cytokines, interleukin-6 (IL-6) and keratinocyte chemoattractant / human growth-regulated chemokine (KC/GRO), over a period of 7 days. Concentration values were normalized to the baseline readings (i.e., serum collected the day of tumor inoculation) and displayed as a value of 1 at T = 0 hours. At 6 hours post-ablation, IL-6 and KC/GRO experienced a significant 27- and 7-fold (p = 0.013 and 0.026, respectively) increase in expression, respectively, compared to that of sham injections (Fig 2A,2B). After 3 days post-ablation, the EC-ethanol injections saw a subsequent drop in IL-6 and KC/GRO expression similar to that of sham injections (14-fold and 3-fold from baseline, respectively, p > 0.10). After 7 days post-ablation, IL-6 expression further decreased towards baseline levels (4-fold from baseline), along with KC/GRO expression (2-fold) decreasing below that of sham injection (3-fold, p > 0.10). The sham injections saw a marginal increase in IL-6 expression after 3 days before dropping to baseline levels after 7 days, while KC/GRO concentrations remained stable throughout the procedure (p > 0.10). The remaining cytokines tested did not exhibit significant differences between treatment groups (IFN-γ, TNF-α, IL-1β, IL-2, IL-4, IL-5, IL-10, IL-12p70) (S1 Fig).

### EC-ethanol treatment induced large regions of tumor necrosis

**Fig 3:**
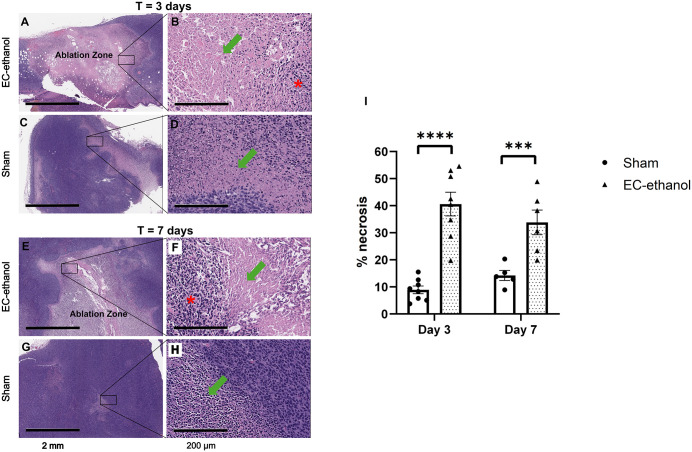
Visualization of the tumor ablation zone induced by EC-ethanol. Representative H&E-stained tumors treated by (A,E) EC-ethanol at 3 and 7 days post-ablation or (C,G) sham treatments at 3 and 7 days post-ablation. Scale bars = 2 mm. Magnified inserts of the necrotic regions at 20x for tumors treated by (B,F) EC-ethanol at 3 and 7 days post-ablation or (D,H) sham treatments at 3 and 7 days post-ablation are shown to illustrate the patterns of coagulative (red star) and liquefactive (green arrow) necrosis. Scale bars = 200 µm. **(I)** Quantification and comparison of the amount of necrosis within the tumor between EC-ethanol and sham treatment groups (n = 5-8). Error bars are standard error of the mean (SEM). *P < 0.10, **P < 0.05, ***P < 0.01, ****P < 0.001.

Fig 3 illustrates the tumor ablation zone induced by EC-ethanol, which is defined by the regions predominantly made visible only by eosin staining (i.e., light pink), compared to the untreated tumor region where hematoxylin staining (i.e., dark purple) is prevalent (Fig 3A-G). The ablation zone for the EC-ethanol treatments at both 3 and 7 days post-ablation displayed patterns of both coagulative necrosis (no intact nuclei, but tissue architecture is still preserved) and liquefactive necrosis (tissue architecture is degraded into a liquified mass) (Fig 3B,F) while the sham treatments only had small pockets of liquefactive necrosis scattered throughout the tumor (Fig 3D,H). Notably, the amount of necrosis from the EC-ethanol treatment (i.e., the ablation zone size) was significantly larger than controls for both time points (41% ± 4% vs. 9% ± 1% at day 3, p < 0.001, and 34% ± 5% vs. 14% ± 2% at day 7, p = 0.0050, for EC-ethanol and sham, respectively) (Fig 3I).

### Cytotoxic T-cells accumulated within ablation zone vicinity

**Fig 4:**
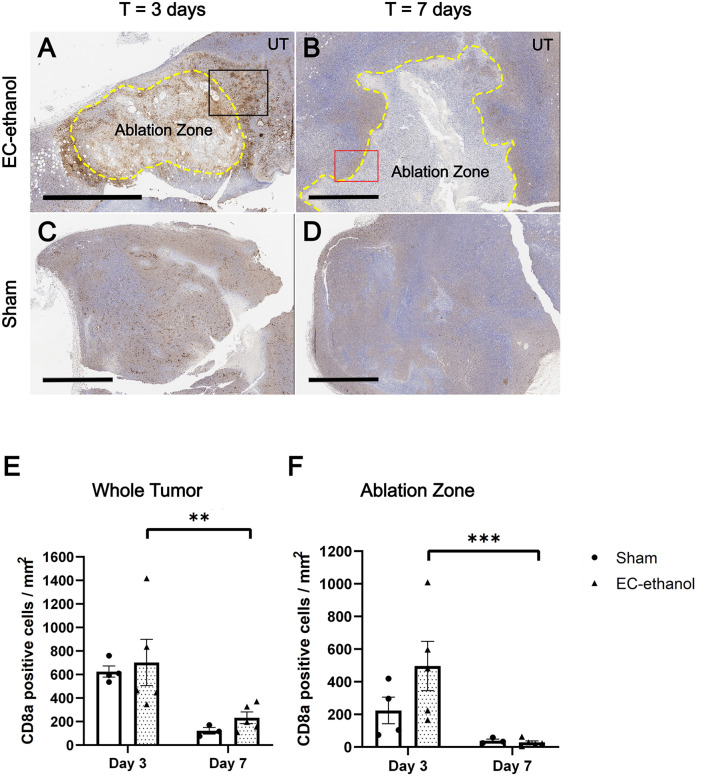
Increased infiltrating cytotoxic T-cells into the ablated tumor after EC-ethanol ablation. Representative IHC-stained tumors for CD8a treated by (A,B) EC-ethanol at 3 and 7 days post-ablation or (C,D) sham treatments at 3 and 7 days post-ablation. UT = untreated tumor. Scale bars = 2 mm. (E) Quantification and comparison of the amount of CD8a + CTLs within the tumor and (F) within the ablation zone only (denoted as the region bordered in yellow dashed lines) between EC-ethanol and sham treatment groups (n = 3-5). *P < 0.10, **P < 0.05, ***P < 0.01, ****P < 0.001.

**Fig 5:**
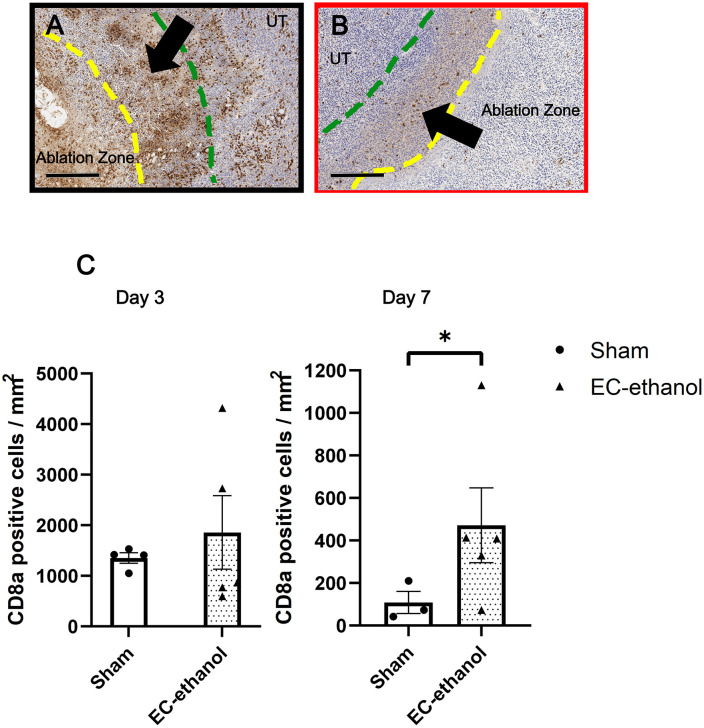
Substantial accumulation of cytotoxic T-cells around the margins of the ablation zone. Closeups of the EC-ethanol ablation zone margins after (A) 3 days (black box) and (B) 7 days (red box), with the margins (region denoted with black arrow and boundary delineated with yellow and green dashed lines) defined as 400 µm out from the ablation zone perimeter. Quantification and comparison of the amount of CD8a + CTLs within the margins at (C) 3 and (D) 7 days post-ablation (n = 3-5). Error bars are standard error of the mean (SEM). Scale bars = 300 µm. *P < 0.10, **P < 0.05, ***P < 0.01, ****P < 0.001.

Fig 4 illustrates the tumor IHC staining results for CD8a, which detects CTLs that infiltrated into the tumor 3 and 7 days post-ablation (Fig 4A-D). Tumors treated with EC-ethanol saw a mild increase in recruitment of CD8a + CTLs at both time points (702 ± 197 and 232 ± 50 positive cells/mm^2^ for days 3 and 7, respectively), compared to those from the sham treatment (625 ± 48 and 122 ± 28 positive cells/mm^2^ for days 3 and 7, respectively), though a significant drop in CTLs after day 7 was observed (p = 0.016) (Fig 4E). The density of CD8a + CTLs within the ablation zone dropped slightly (496 ± 151 positive cells/mm^2^) compared with that of the entire tumor at day 3 (Fig 4F), though there were significantly fewer CTLs present after day 7 (28 ± 10 positive cells/mm^2^, p = 0.0079). The sham treatment saw a similar decrease of CTLs from 224 ± 82 positive cells/mm^2^ down to 39 ± 10 positive cells/mm^2^. When examining the margins surrounding the ablation zone induced by EC-ethanol (Fig 5A-B), there was a 1.4-fold (1858 ± 727 positive cells/mm^2^) increase in CD8a + CTL population density at day 3, compared to sham treatment (1353 ± 105 positive cells/mm^2^, Fig. 5C, p > 0.10). An even larger 4.3-fold (471 ± 176 positive cells/mm^2^) increase in CD8a + CTL population density was observed at day 7 compared to sham treatment (109 ± 52 positive cells/mm^2^, p = 0.071, Fig 5D).

### Increased cytotoxic T-cells within the contralateral tumor after 7 days post-ablation

**Fig 6:**
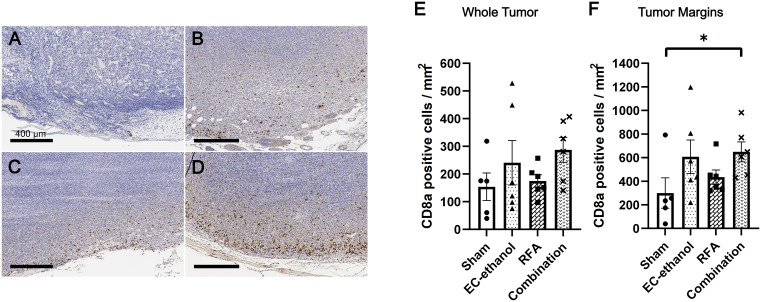
Increased infiltrating cytotoxic T-cells in the contralateral tumor after ablative treatments. Representative IHC-stained contralateral tumors for CD8a treated by (A) sham, (B) EC-ethanol, (C) RFA, or (D) combination (EC-ethanol + RFA) at 7 days post-ablation. Scale bars = 400 µm. Quantification and comparison of the amount of CD8a + CTLs within (E) the entirety of the contralateral tumor and (F) within the margins of the tumor periphery (n = 5-6). Error bars are standard error of the mean (SEM). *P < 0.10, **P < 0.05, ***P < 0.01, ****P < 0.001.

Fig 6 illustrates the contralateral tumor IHC staining results for CD8a after 7 days post-ablation (Fig 6A-D). Primary tumors treated with either EC-ethanol or RFA resulted in an increased number of CD8a + CTLs within the contralateral tumor (241 ± 80 and 175 ± 23 positive cells/mm^2^ for EC-ethanol and RFA, respectively) compared to that from the sham treatment (154 ± 50 positive cells/mm^2^, p > 0.10) (Fig 6E). The combination treatment also saw an increased number of recruited CTLs in the contralateral tumor, at 287  ± 45 positive cells/mm^2^ (p > 0.10). All treatment groups saw a majority of the CTLs accumulating along the margins of the tumor periphery, which saw a higher density of CTLs within this region compared to that from the sham treatment (Fig 6F). There was a 2-fold (607 ± 142 positive cells/mm^2^) and 1.5-fold (435 ± 61 positive cells/mm^2^) increase in CTL population density after EC-ethanol and RFA treatment, respectively, compared to sham treatment (300 ± 129 positive cells/mm^2^) (p > 0.10). Notably, the combination treatment saw a significant 2.2-fold increase in CTL population density (649 ± 84 positive cells/mm^2^), compared to that of sham treatment (p = 0.094).

## Discussion

Ethanol ablation possesses some advantages over other locoregional ablative therapies and may potentiate antitumor immune effects. However, it suffers from ethanol leakage, which adversely impacts ethanol distribution within the tumor and diminishes treatment effectiveness. Adding EC to the ethanol injectate results in the formation of an EC-ethanol gel depot to form within the tumor, preventing ethanol leakage and improving tumor ablative efficacy [22]. This study implemented both single and bilateral flank CRC murine models to explore the ability of EC-ethanol to induce both local and systemic antitumor immune effects, respectively. These effects were evaluated by measuring cytokine serum levels at multiple time points following intratumoral EC-ethanol injection and tumor immunohistochemistry analysis of CD8a.

Ablative therapies often induce the secretion of cytokines, which promote immune cell infltration and antigen recognition [37]. The significantly increased expression of IL-6 and KC/GRO cytokines within the first 3 days post-ablation suggests that EC-ethanol induces an inflammatory response (Fig 2), which is consistent with cytokine profiles induced by other ablative therapies within the first week [38,39]. IL-6 recruits innate immune cells such as neutrophils and macrophages [40,41]; it also plays a key role in the adaptive immune response including inducing the differentiation of naïve T cells into either helper or cytotoxic cells [42]. KC/GRO (a chemokine analogous to CXCL1) is a neutrophil chemoattractant and also a mediator for angiogenesis [43,44]. The subsequent drop in IL-6 and KC/GRO levels after 3 and 7 days suggest a shift from the inflammation phase to the proliferation phase [45,46], which may correspond to the onset of an adaptive immune response and CTL recruitment to the ablation zone and its margins (Fig 5). This early elevation in cytokines by EC-ethanol ablation is consistent with a previous report demonstrating an elevation in IL-6 and KC/GRO serum levels 6 hours after cryoablation of mouse CT26 tumors followed by a drop in IL-6 and KC/GRO serum levels after 3 days [25]. A clinical study also demonstrated elevations in IL-6 within 48 hours of thermal ablation [47]; however, increased IL-6 levels correlated with decreased overall survival in 99 patients with CRC cancers [48,49]. A study in CT26-bearing mice showed that upregulated CXCL1 levels after 2 weeks correlated with exacerbated tumor growth [50]. Thus, while EC-ethanol ablation may suffice in generating strong antitumoral signals comparable to those of established modalities to treat CRC, further studies are merited to ensure EC-ethanol treatments will result in improved cancer patient survival. Modulating the concentrations of pro-inflammatory cytokines such as IL-6 and KC/GRO may enhance the therapeutic benefits of EC-ethanol ablation on treating CRC.

The primary mechanism of ethanol ablation is thought to be the induction of coagulative necrosis due to ischemia [51,52]. Specifically, it has been shown to induce vascular occlusion and subsequently disrupt tumor blood supply. Here, tumor regions treated with EC-ethanol contained both coagulative and liquefactive necrosis while sham treatments only had small pockets of liquefactive necrosis scattered throughout the tumor (Fig 3). Coagulative necrotic regions contained cells lacking nuclei but maintained tissue structural architecture. Conversely, regions of liquefactive necrosis lacked consistent architecture with lysed cells, debris, and clear space. Liquefactive necrosis was more prominent within the center of the EC-ethanol ablation zone, while coagulative necrosis tended to be prevalent near the edge of the ablation zone. These observations corroborate the results from a study where a combination therapy of EC-ethanol and photodynamic therapy resulted in liquefactive necrosis within the center of the depot and coagulative necrosis along the perimeter of the depot after 7 days post-ablation [30].

Necrotic cell death induced by EC-ethanol also typically results in the release of damage-associated molecular patterns, which include tumor-specific antigens that can be picked up by antigen-presenting cells and presented to prime the adaptive immune system for long-term immunity [53], specifically the priming of T-cells. The presence of CD8a + T-cells within the tumor is an important indicator of potential antitumor effects after ablation. Following intratumoral EC-ethanol injection, there was a mild increase in CD8a + CTLs compared to that of sham throughout the 7 day post-ablation period (Fig 4E). The majority of these cells were situated within the ablation zone or around its immediate margins (Fig 4F, 5D). The ablation margins, defined as the interface between intact tumor and the ablation zone, was comprised of a thick band of densely packed CTLs at day 3 (Fig 5C) before thinning to a more well defined perimeter along the edge of the ablation zone at day 7 (Fig 5D). This signifies strong accumulation of CTLs within the EC-ethanol depot interface and suggests prolonged treatment effects to the surrounding untreated tumor regions. The increased presence of CTLs into the tumor corroborates previous results in which EC-ethanol treatment in a murine chemically-induced triple-negative breast cancer model recruited CD3 + general T-cells into the tumor after 30 days post-ablation [24]. This study further expands on this observation by specifically characterizing CD8a + CTLs within the tumor margin and quantifying their cell population, which is integral in characterizing the cytotoxicity and antitumor efficacy of EC-ethanol ablation. Future work will characterize the co-localization of CTLs with other immune infiltrates (e.g., T-helper cells, B-cells, macrophages) to elucidate the interplay behind the immunomodulatory properties of EC-ethanol ablation, along with the expression of inhibitory receptors and ligands (e.g., PD-1, PD-L1) to identify potential targets for combinatorial therapeutic strategies with EC-ethanol to ablate CRC tumors.

The abscopal effect is predicated by the presence of antitumor effects on the contralateral tumor after local ablation of the primary tumor. In the present study, EC-ethanol treatment yielded a comparable number of CTLs in contralateral tumors to that observed with RFA on day 7 (Fig 6F), suggesting that EC‑ethanol is similarly effective to RFA in promoting CTL recruitment to contralateral tumor sites. Previous studies have demonstrated that combining percutaneous ethanol ablation with RFA confers superior local tumor control and improved long-term survival in patients with liver tumors when compared to RFA alone [34,35]. Thus, to initially assess the antitumor effects induced by combinatorial ablative therapies, we combined EC-ethanol ablation and RFA together in one treatment regimen on the primary tumor and evaluated any improvement in quality of the immune response on the contralateral tumor. There was a 2-fold increase in CD8a + CTLs in the contralateral tumor for the combination treatment group compared to that of the sham, along with a 1.6- to 1.2-fold increase compared to EC-ethanol and RFA monotherapies, respectively (Fig 6E). Additionally, there was a significant 2.2-fold increase in CTLs within the margins of the contralateral tumor after combination therapy compared to that of the sham (Fig 6F), while there were no significant differences observed between the other treatment groups (Fig 6F), suggesting synergistic effects between EC-ethanol and RFA treatments. Future work will investigate increasing the dosage of the treatments (e.g., larger volume of EC-ethanol, multiple injections on a single tumor) to achieve a therapeutic dosage and then assess their antitumor efficacy, and implement a longitudinal study to evaluate the ability of EC-ethanol-based treatments over a longer period of time to improve survival rates.

This study had limitations. First, subcutaneous CRC tumors were inoculated in the flank of mice. Future studies will include assessing the antitumor effects of EC-ethanol in an orthotopic CRC model [54,55] to better recapitulate the native tumor microenvironment of CRC and its potential impact on immune effects. Second, we assessed immune effects at only two time points post-treatment, and evaluation at later stages was precluded by the inherent aggressive growth of tumors and the implementation of subtherapeutic dosages to examine immune cell populations in both treated and untreated regions within the tumor. Given that antitumor immunity is a dynamic process, future studies will incorporate additional time points, increased therapeutic dosages, and may employ slower-growing tumor models to enable a more comprehensive assessment of the immune responses elicited by EC‑ethanol treatment. Survival studies will also be conducted to compare and contrast the efficacy of EC-ethanol treatments with RFA and other standard-of-care modalities. Lastly, due to the highly immunogenic nature of the CT26 tumor model, the differences in antitumor effects and immune cell recruitment between EC-ethanol and sham treatments may be partially masked. Future studies will obtain data on immune cell populations in the tumor before injection.

## Conclusions

EC-ethanol ablation generated local and systemic antitumoral effects on CRC tumors over a period of 7 days. The increased expression of IL-6 and KC/GRO cytokines within the first 6 hours post-ablation indicated a strong pro-inflammatory immune response was elicited by EC-ethanol treatment. Over a period of 7 days, a gradual drop in cytokine expression was complemented with the large recruitment of CD8a + CTLs into the vicinity of the ablation zone within the primary tumor. These changes in cytokine expression and cell populations indicated a shift towards an adaptive immune response to stunt CRC tumor growth. The contralateral tumors also saw an increase in CTL infiltration after EC-ethanol ablation, signifying the capability of EC-ethanol to potentiate abscopal effects. Altogether, these results suggest that EC-ethanol potentiates antitumoral effects in CRC tumors and is a strong therapeutic candidate for treating CRC patients worldwide.

## Supporting information

S1 Fig Pro-inflammatory cytokine expression induced by EC-ethanol ablation. Normalized concentration values of cytokine profiles induced by EC-ethanol and sham treatments over a period of 7 days (n = 8−10). IL-4 and IL-12p70 did not generate any quantifiable readings at any time point. Error bars are standard error of the mean (SEM). *P < 0.10, **P < 0.05, ***P < 0.01, ****P < 0.001.
(TIF)
