# Supplementary figures and images for: Antitumoral immunity induced by gel ethanol ablation to treat unresectable colorectal cancer metastases in the liver
Source: PLoS One. 2026 Apr 22;21(4):e0347625. doi: 10.1371/journal.pone.0347625 (PMC13102203; doi:10.1371/journal.pone.0347625)

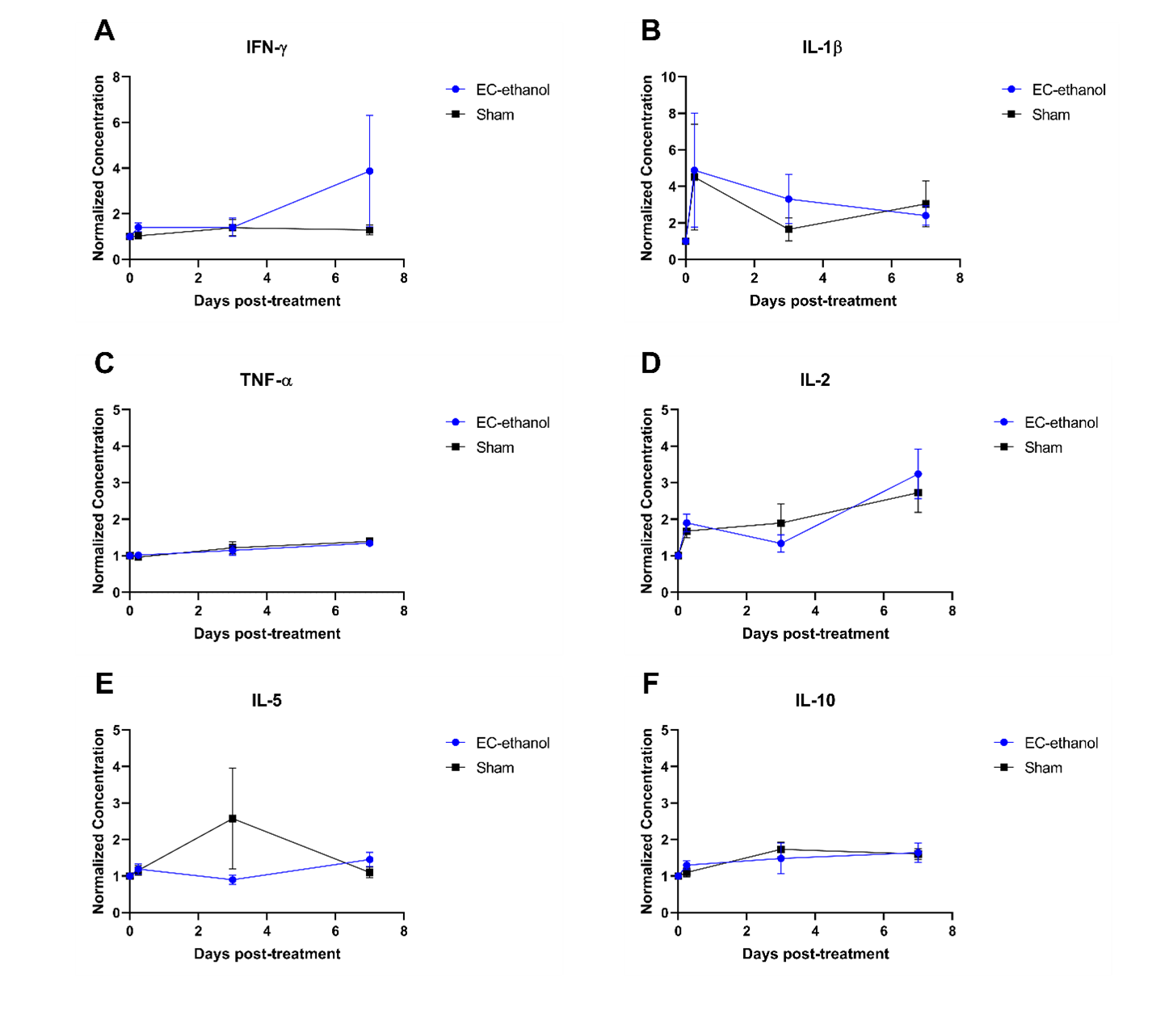

Supplement: S1 Fig — Normalized concentration values of cytokine profiles induced by EC-ethanol and sham treatments over a period of 7 days (n = 8−10). IL-4 and IL-12p70 did not generate any quantifiable readings at any time point. Error bars are standard error of the mean (SEM). *P < 0.10, **P < 0.05, ***P < 0.01, ****P < 0.001. (TIF) [file pone.0347625.s001.tif]
